# Effects of high-frequency hyperthermia on the elastic modulus of the lumbar muscle in female athletes with low back pain: A randomized crossover trial

**DOI:** 10.1097/MD.0000000000038011

**Published:** 2024-04-26

**Authors:** Takayuki Miyamori, Masashi Aoyagi, Taiki Saito, Yuki Masui, Yoshihiko Ishihara, Yu Shimasaki, Masafumi Yoshimura

**Affiliations:** a Department of Physical Therapy, Faculty of Health Science, Juntendo University, Tokyo, Japan; b Juntendo Administration for Sports, Health and Medical Sciences, Juntendo University, Tokyo, Japan; c Rehabilitation Center, Oyumino Central Hospital, Chiba, Japan; d Graduate School of Health and Sports Science, Juntendo University, Chiba, Japan; e School of Science and Technology for Future Life, Tokyo Denki University, Tokyo, Japan; f Department of Health and Sports Science, Faculty of Health and Sports Science, Juntendo University, Tokyo, Japan.

**Keywords:** athletes, back muscles, diathermy, elastic modulus, ultrasonography

## Abstract

**Objective:**

To investigate the effects of capacitive and resistive monopolar radiofrequency (CRMF) on the shear elastic modulus of the multifidus and erector spinae muscles in female athletes with low back pain (LBP) and a history of LBP.

**Design:**

Randomized crossover trial.

**Setting:**

Academic institution.

**Participants:**

Twenty female university athletes with LBP or a history of LBP were included.

**Interventions:**

All participants received CRMF, hotpack, and sham (CRMF without power) in a random order on the right side of the lumbar region. More than 2 days were allocated between the experiments to eliminate any residual effects.

**Main outcome measures:**

The shear elastic moduli of the right multifidus and erector spinae were evaluated in the prone (rest) position while sitting with 35° trunk flexion (stretched) using shear wave ultrasound imaging equipment. The moduli were measured before, immediately after, and 30 minutes after the intervention.

**Results:**

Repeated-measures 2-way analysis of variance and post hoc analysis showed that the moduli of the CRMF group were significantly lower than those of the sham group in the stretched position immediately after intervention (*P* = .045). This difference diminished 30 minutes after the intervention (*P* = .920).

**Conclusions:**

CRMF can be used to reduce the shear elastic modulus of the multifidus muscle in the short term. Further studies are warranted to determine how to provide longer effects.

**Trial registration:**

None.

## 1. Introduction

Low back pain (LBP) is 1 of the most common injuries experienced by athletes, and female athletes are more likely to develop LBP than male athletes,^[1]^ possibly because of their anatomical characteristics, smaller muscle mass, and lower bone densities.^[2]^ In addition to the high prevalence of LBP. It has been reported that the 1-year reoccurrence rate of LBP is between 24% and 54% in patients with a history of LBP.^[3,4]^ Although many athletes have LBP, which can negatively affect their athletic performance,^[5]^ the optimal treatment modality has not been elucidated.

There is some empirical evidence that patients with LBP and a history of LBP may have altered muscle structure, function, and properties in the lower back.^[6–10]^ In previous research, smaller size and decreased electromyographic activity of the multifidus in patients with LBP compared with asymptomatic participants have been reported,^[6,7]^ while increased electromyographic activity of the erector spinae in participants with LBP and a history of LBP compared with those without an episode of LBP was found.^[8]^ As for biomechanical properties, the shear elastic moduli, namely stiffness, of the multifidus and erector spinae, which are evaluated by shear wave elastography (SWE), is higher in individuals with LBP or a history of LBP than in those without LBP.^[9,10]^ The increased stiffness in the multifidus and erector spinae may be a reason behind LBP, as it could induce ischemic pain^[10]^ and maybe a reason for the recurrence of LBP, as stiff muscles could be prone to injury.

In clinical practice, thermal therapy is often employed in the treatment of LBP, as it is believed to reduce pain by increasing tissue temperature and blood flow.^[11]^ Although many physical agent modalities are used in thermal therapy, capacitive and resistive monopolar radiofrequency (CRMF), which is a diathermy treatment, has attracted increasing attention. CRMF delivers radiofrequency energy at 448 kHz, while traditional diathermy devices using radiofrequency, such as shortwave therapy, operate at 27.12 MHz.^[12]^ Therefore, CRMF is safer than traditional devices because it does not cause excessive heat generation.^[13]^ CRMF is categorized as deep thermal therapy, while other traditional thermal therapy devices, including hotpacks, are classified as superficial thermal therapies.^[13]^ In the treatment of chronic LBP, Tashiro et al^[14]^ reported that CRMF adjunct to exercise was more effective in reducing pain than exercise alone. As CRMF can improve vasodilation, the authors suggested that the thermal effect of CRMF could suppress ischemia and spasticity in the lumbar region, consequently reducing pain. However, whether CRMF alters muscle properties, which can be altered in patients with LBP and a history of LBP,^[9,10]^ has not been fully elucidated. Understanding the mechanism of the possible effects of the physical agent modality is important in decision-making pertaining to treatment, as the sources of pain in these patients can be diverse. Therefore, this study aimed to investigate the effects of CRMF on stiffness, which was evaluated using the shear elastic modulus of the multifidus and erector spinae muscles, in female athletes with LBP or a history of LBP.

## 2. Methods

### 2.1. Participants

Recruitment was conducted targeting female university athletes utilizing the university athletic training room at Juntendo University. The inclusion criteria were engaging in daily sports activities, suffering from LBP, or having a history of LBP within 6 months. LBP was defined as any pain that occurred between the gluteal folds inferiorly and along the border of the 12^th^ rib superiorly^[15]^ rated more than 1 of 10 at rest and 3 of 10 when playing a sport on the numerical rating scale but did not prevent the participants from being fully involved in team practice and games. The existence and experience of LBP were investigated using the original questionnaire. The exclusion criteria were patients with severe LBP that prevented them from participating in sports or diseases and injuries of the nervous, respiratory, and circulatory systems; thrombophlebitis; history of spinal surgery; wound and burn injury at the lumbar area; implantable electronic devices; and sensory disorders. All participants underwent the test for the sense of temperature at the lumbar skin with test tubes filled with water at different temperatures (±0.5°C): warm, 45°C; neutral, 35°C; and cold, 25°C.^[16]^ The examiner placed all test tubes were placed randomly on the right side of the lumbar spine while participants were in the prone position. Subsequently, participants were required to indicate the order of the applied temperatures. Participants who correctly distinguished between the 3 temperatures were enrolled in the study.

### 2.2. Study design

**Figure 1.**
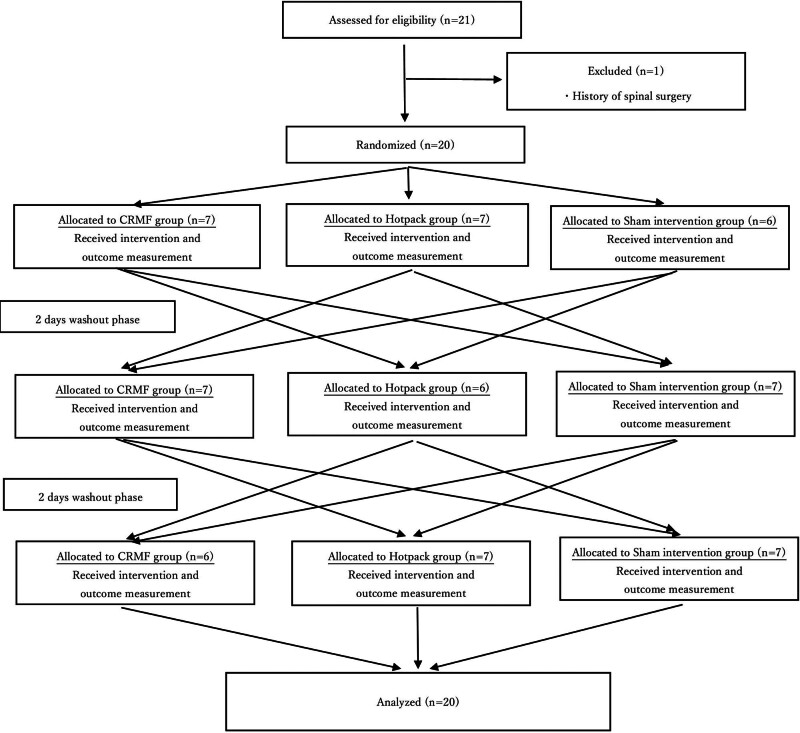
Flow diagram for the current crossover trial.

This study was conducted as a randomized crossover trial to evaluate the effects of CRMF on multifidus and erector spinae stiffness at the Juntendo University Sports Clinic from June to August 2022 and reported according to the Consolidated Standards of Reporting Trials statement.^[17]^ Participants were randomly assigned to 1 of 3 interventions: CRMF, hotpack, and CRMF without power (sham) in the first experiment. Subsequently, they were randomly assigned to 1 of the remaining 2 interventions in the second experiment and received the last intervention in the third experiment (Fig. 1). Experienced physical therapists who were not involved in any of the outcome evaluations administered the intervention. The study protocol did not change after trial commencement.

### 2.3. Randomization and masking

Computer-based block randomization was performed to ensure an unbiased allocation sequence (Fig. 1). Although participants were blinded to the intervention allocation (CRMF or sham intervention), they were not blinded to hotpack allocation. A researcher who was blinded to the intervention allocation evaluated the participants. Recruitment and randomization were performed by a chief researcher who was not involved in the intervention and evaluation.

### 2.4. Interventions

The intervention was applied to the right lumbar region, which was longitudinally from the 12th thoracic vertebra to the 5th lumbar vertebra and horizontally from the spine to 15 cm to the right, irrespective of pain location. The intervention region was defined as increased stiffness in this region in participants with LBP and history of LBP, irrespective of pain location, as reported in our previous study.^[18]^

**Figure 2.**
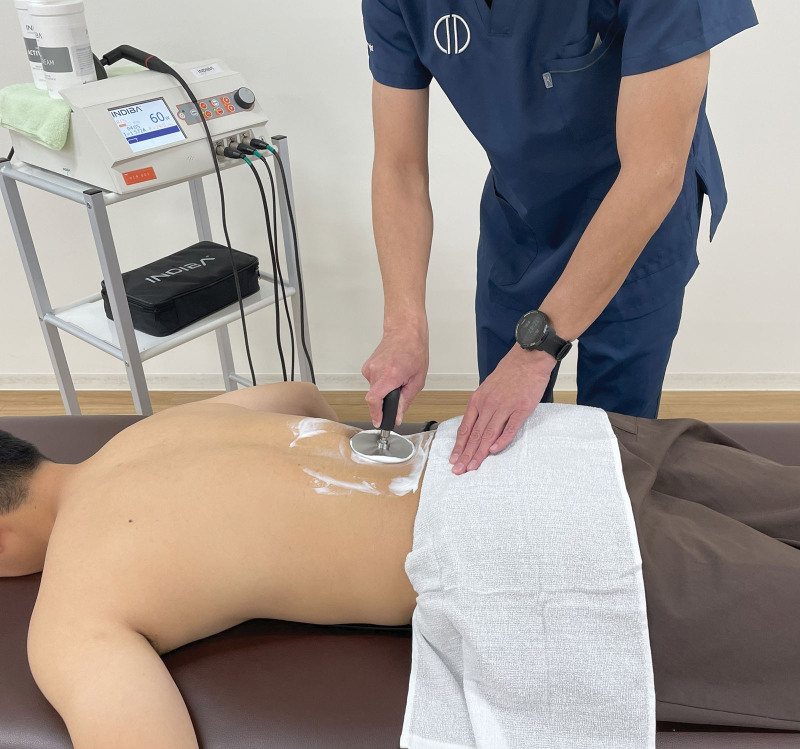
Capacitive and resistive monopolar radiofrequency intervention application.

Indiba activ HCR801 (INDIBA, Barcelona, Spain) operating at a frequency of 448 kHz was used for CRMF intervention (Fig. 2). This device delivered continuous radiofrequency energy in 2 modes, capacitive (CAP) and resistive (RES), through metallic active electrodes. Because the CAP electrode is coated with polyamide, it induces external heat near the skin, whereas the RES electrode can generate heat in the deeper area by delivering radiofrequency energy through the body into the inactive electrode as it is uncoated. A metallic plate (200 × 260 mm) was used as the inactive electrode. The participants were placed in the prone position, while the inactive electrode was placed on the abdomen to avoid contact with the bone and reduce the risk of burns. The irradiation intensity started at 27 W for CAP and 30 W for RES, and was increased by 1 W every 30 seconds until the participants complained of 6 or 7 of the 10 thermal sensations (0, none; 10, worst possible thermal sensing) according to the manufacturer’s instructions. The total irradiation time was 15 minutes, with 5 minutes for CAP and 10 minutes for RES.^[13]^ Consequently, the intensity ranges were between 27 and 51 W for CAP and between 30 and 60 W for RES. During the intervention, a manufacturer-supplied conductive cream was used as the coupling medium and the electrodes were manipulated in circular motion along the target region.

For the hotpack intervention, an ore hotpack (PHYSIOPACK PPS-01; SAKAI Medical Co., Tokyo, Japan) heated to 80°C was used. A hotpack was then placed in the target region for 15 minutes. For the Sham intervention, the same procedure as that used for the CRMF intervention without power was performed.

### 2.5. Measurement

Back muscle stiffness was evaluated using shear wave ultrasound imaging equipment (Applio300; Canon Medical Systems, Tochigi, Japan), based on a previous study.^[18]^

The shear elastic moduli of the right multifidus and erector spinae were evaluated in 2 positions: the rest position, in which the participants were placed in the prone position on the bed, and the stretch position, in which the participants sat on a chair with their trunks flexed at a 35° angle. These 2 positions were selected because the shear elastic modulus of the muscle can change when it is stretched. The stretch position was determined by stretching both the multifidus and erector spinae muscles based on anatomical considerations. In each position, the participants were asked to relax as much as possible while the pillow and cushion were placed on the head and abdomen, respectively, to reduce the muscle activity of the lower back.^[18]^

Identification of the multifidus and erector spinae was performed using the B-mode of SWE and a linear array ultrasound probe (PLT-705BT, Cannon Medical Systems, Tochigi, Japan) with frequency ranging between 4.0 MHz and 14.0 MHz. The stiffness of the right multifidus was measured 2 cm lateral to the middle region between the L4 and L5 spinous processes, and that of the right erector spinae was measured 7 cm lateral to the L3 spinous process (iliocostalis lumborum).^[18,19]^ The details of the measurements are described in our previous study.^[18]^ The reliability and validity of assessing lumbar muscle stiffness have been established.^[9,18,20,21]^

To measure the shear elastic modulus, 3 circular regions of interest (ROIs) with a diameter of 10 mm were set within the SWS box (20 mm × 20 mm), with 1 placed at the top center of the box and the others at the bottom.^[18]^ All the SWE images were saved during terminal exhalation to stabilize color distribution within the muscle. The measurements were repeated thrice for each muscle at each position. The mean shear elastic modulus values in each ROI were calculated from 3 trials and the mean values of the 3 ROIs were subsequently obtained as representative values. The shear elastic modulus (kPa) was computed based on the shear wave velocity within the tissue (V) and the density of the muscle (p) according to the following formula: shear elastic modulus (kPa) = pV^2^, which suggests that the higher the shear wave velocity, the higher the shear elastic modulus, and thus, the stiffer the muscle. These outcomes did not change after the trial commencement.

### 2.6. Procedures

The shear elastic modulus was measured before (Pre), immediately after (Post), and 30 minutes after (Post-30) intervention. After the postmeasurement period, the participants were instructed to lie in the supine position in a bed and rest with their knees bent for 30 minutes. As this study design was a crossover trial, a minimum gap of 2 days between experiments was allowed to eliminate the residual effects of previous experiments.^[16]^

### 2.7. Statistical analyses

SPSS software (version 20.0; IBM Japan, Tokyo, Japan) was used for all the statistical analyses. To examine the effects of the intervention and changes over time, a 3 × 3 (intervention × time) repeated-measures 2-way analysis of variance was conducted. Mauchly sphericity test was performed, and when the sphericity assumption was rejected (*P* > .05), the data were corrected for degrees of freedom using Greenhouse–Geisser epsilon correction. When the interaction was significant, multiple comparisons were conducted using Dunnett post hoc test. Statistical significance was set at *P* < .05. As the sample size was not calculated before recruitment, posthoc power analysis was performed to confirm the probability of a type-II error using G*power 3.1.9.7 software,^[22]^ where statistical significance was observed.

### 2.8. Ethical considerations

Written informed consent was obtained from each participant prior to enrollment. This study was approved by the Ethics Committee of the University (approval number: 2021-54).

## 3. Results

This study included 20 out of 21 female student athletes who engaged in football or softball were included in this study (Fig. 1). The demographic data are shown in Table 1, and the changes in the shear elastic modulus of the multifidus and erector spinae muscles over time for each intervention are shown in Table 2. Repeated-measures 2-way analysis of variance showed a significant interaction in the multifidus in the stretching position (F4,76 = 3.832; *P* = .007; η^2^ = 0.168, Power = 0.999). Post hoc analysis showed that the shear elastic modulus of the multifidus in the stretching position post-CRMF was significantly lower than that after sham (*P* = .045), although this difference was not statistically significant at Post-30 (*P* = .920). No adverse reactions related to the hotpack or CRMF were reported.

**Table 1:** Descriptive statistics of the participants.

	Participants with LBP or history of LBP
	(n = 20)
Age (yr)	19.3 ± 1.2
Height (cm)	161.4 ± 4.6
Weight (kg)	56.8 ± 4.8
BMI	21.8 ± 1.8
Sex (male/female)	0/20

Data are presented as mean ± standard deviation.

BMI = body mass index, LBP = low back pain.

**Table 2:** Results of repeated-measures 2-way analysis of variance and post hoc analysis.

	Muscles	Time	Participants (n = 20)			Repeated-measures 2-way ANOVA				Post hoc analysis	
			Intervention			Interaction (intervention × time)				Dunnett test	
			CRMF	HP	Sham	F	*P*	η^2^	Power	Comparison	*P*
Rest position	Miltifidus (kPa)	Pre	35.2 ± 7.8	35.2 ± 10.0	35.3 ± 11.6						
		Post	27.4 ± 10.4	35.2 ± 9.0	34.3 ± 12.4	1.910	.117				
		Post-30	24.6 ± 9.6	31.7 ± 10.2	30.0 ± 8.5						
	Erector spinae (kPa)	Pre	14.4 ± 4.8	14.5 ± 5.0	14.3 ± 5.9						
		Post	11.8 ± 3.1	13.8 ± 5.4	13.7 ± 4.1	0.866	.488				
		Post-30	12.9 ± 3.4	13.5 ± 5.8	14.2 ± 5.5						
Stretch position	Miltifidus (kPa)	Pre	85.0 ± 11.3	77.3 ± 17.5	82.4 ± 19.6						
		Post	71.7 ± 15.9	76.6 ± 17.6	79.6 ± 20.7	3.832	.007**	0.168	0.999	CRMF vs HP CRMF vs Sham	.266 .045*
		Post-30	72.2 ± 20.2	79.2 ± 18.2	77.8 ± 18.1					CRMF vs HP CRMF vs Sham	.348 .920
	Erector spinae (kPa)	Pre	19.9 ± 7.8	22.0 ± 12.9	22.2 ± 14.0						
		Post	16.7 ± 7.0	23.2 ± 16.0	21.6 ± 12.7	0.663	.620				
		Post-30	16.4 ± 7.2	21.6 ± 14.7	20.3 ± 12.3						

Data are presented as mean ± standard deviation.

ANOVA = analysis of variance, CRMF = capacitive and resistive monopolar radiofrequency, HP = hotpack.

*< .05.

** <.01.

## 4. Discussion

The current results showed that CRMF had an immediate effect on significantly reducing the shear elastic modulus of the multifidus muscle during stretching in female athletes with LBP or a history of LBP. Tashiro et al^[13]^ reported that deep lumbar tissue with a depth of 10 to 20 mm subcutaneously had 3 to 4°C higher temperatures immediately after 15 minutes of CRMF, which could increase the extensibility of collagen fibers.^[23]^ Moreover, the results showed an improvement in blood flow immediately after CRMF, which is thought to be a result of the suppression of sympathetic nerve activity and an increase in parasympathetic nerve activity caused by thermal stimulation.^[13]^ These changes in the autonomic nervous system can result in reduced stiffness of the multifidus after CRMF, as the parasympathetic nerve could relax the muscles. Previous studies have reported that the shear elastic modulus of the multifidus is higher in individuals with LBP or a history of LBP than in healthy individuals, which is thought to be due to muscle spasms.^[9,10]^ Therefore, the current results suggest that CRMF may reduce muscle spasms in the multifidus muscle.

Although a significant change was observed in the multifidus stiffness after CRMF, no change was observed in the erector spinae stiffness. There are several possible explanations for this finding. First, CRMF could affect deeper tissues rather than those that are less shallow, considering the characteristics of the radiofrequency energy at 448 kHz, which penetrates into the former.^[13]^ Second, energy absorption may differ between muscles, as the shear elastic moduli are different. We previously reported that female athletes with LBP or a history of LBP had significantly higher multifidus stiffness than healthy athletes, with no differences in the characteristics of the erector spinae.^[18]^ In the current study, the multifidus exhibited higher stiffness than the erector spinae before the intervention (Table 2). It is possible that the influence of the thermal effect of CRMF may differ depending on the differences in muscle properties. Another explanation relates to the measurement position. Masaki et al^[24]^ evaluated the positions that can stretch the multifidus and erector spinae and reported that the multifidus can be stretched the most during seated trunk flexion, whereas the erector spinae can be stretched the most during a combination of seated trunk flexion and contralateral lateral bending. In our study, the stretch position was defined as 35° of trunk flexion only in the sitting position; therefore, in this measurement position, the multifidus could be effectively stretched, whereas the erector spinae could not. Therefore, changes in shear elastic modulus are more likely to occur in the multifidus than in the erector spinae.

The shear elastic modulus of the multifidus muscle changed immediately after CRMF, but disappeared after 30 minutes. Kumaran et al^[16]^ reported that CRMF to the thigh induced an increase in skin temperature, which continued for 45 minutes after the intervention. Similarly, Tashiro et al^[13]^ reported that the thermal effects of CRMF on the lumbar region were still present 50 minutes after the intervention. As the association between body temperature and the shear elastic modulus of soft tissues has not been established, it is unclear whether the thermal effects disappeared 30 minutes after CRMF in this study. However, it is possible that the intensity and duration of CRMF irradiation were insufficient to induce long-term effects in the study participants. In the current study, the irradiation intensity of CRMF was adjusted based on subjective heat sensation according to the manufacturer’s safety instructions, which was also used in a previous study.^[13]^ The difference between the studies was the sex of the participants; the current study employed female individuals, while Tashiro et al’s study included male individuals. Although female individuals have been reported to have a higher fat mass than male individuals, the body fat percentage is negatively correlated with thermal onset when the participants stated that they felt heat,^[16]^ suggesting that the higher the body fat percentage of the participants, the more easily they felt the thermal sensation. Thus, the irradiation intensity of CRMF may have been lower in the current study than that in the previous study. Therefore, owing to the insufficient irradiation intensity of CRMF, changes in the multifidus muscle may not have continued for 30 minutes.

This could explain why no change in multifidus stiffness was observed in the remaining position. If the amount of irradiation of the CRMF was sufficient to induce changes in stiffness in the resting position, the changes in stiffness in the stretching position would have been sustained for a long period of time. Navarro-Ledesma et al^[25]^ reported that 9 CRMF interventions performed 3 times a week resulted in a reduction in supraspinatus stiffness that lasted for 1 week. Tashiro et al^[14]^ reported that a total of 10 CRMF interventions combined with exercise therapy resulted in a decrease in pain intensity in patients with LBP, and the effects lasted for 1 month.

This study has some limitations. First, the effect of CRMF on LBP and the association between the decrease in shear elastic modulus and LBP are unknown because the pain intensity was not assessed. However, individuals with LBP or a history of LBP have increased lumbar muscle stiffness, which may be due to the presence of muscle spasms. Muscle spasm (increased stiffness) can cause local ischemia, leading to the development of LBP,^[10]^ and CRMF can relieve stiffness through thermal effects and can be used as a treatment for patients with LBP caused by muscle spasms. Second, the evaluation and intervention were conducted on the right side of the lumbar region, regardless of symptom location. Therefore, it was unclear whether the changes observed in the current study could be obtained when the same intervention was applied to the symptomatic site. Although some morphological differences may exist between the left and right sides, we believe that these differences are unlikely to affect current thermal therapy results. Third, we did not objectively evaluate the thermal effects of CRMF, such as the total amount of energy used during CRMF irradiation or local temperature. As the irradiation intensity of the CRMF was adjusted based on subjective sensation, the total amount of irradiation and the thermal effect may vary among participants. Further studies assessing the effects of CRMF on pain intensity and investigating the long-term effects of CRMF are necessary to strongly recommend its use in female athletes with LBP.

## 5. Conclusion

To the best of our knowledge, this study is the first to examine the effects of CRMF on the shear elastic modulus in the multifidus and erector spinae muscles in female university athletes with LBP or a history of LBP compared with the effects of hotpack and sham treatment. The results demonstrated that CRMF reduced the shear elastic modulus of the multifidus in the stretched position in the short term, whereas the hotpack did not affect that of the lumbar muscles. Although further research is needed to determine its long-term effects, CRMF can be used as a treatment for female athletes with LBP.

## Acknowledgments

We would like to thank the Research Project of the Institute of Health and Sports Science & Medicine at Juntendo University for their valuable assistance in conducting this study. We would also like to thank Editage (www.editage.com) for the English language editing.

## Author contributions

**Conceptualization:** Takayuki Miyamori, Yu Shimasaki.

**Formal analysis:** Masashi Aoyagi.

**Funding acquisition:** Takayuki Miyamori.

**Methodology:** Masashi Aoyagi, Yuki Masui, Yoshihiko Ishihara.

**Project administration:** Taiki Saito.

**Validation:** Masashi Aoyagi.

**Visualization:** Taiki Saito.

**Supervision:** Masafumi Yoshimura.

**Writing – original draft:** Takayuki Miyamori.

**Writing – review & editing:** Takayuki Miyamori, Masashi Aoyagi, Yuki Masui, Yoshihiko Ishihara, Masafumi Yoshimura.
